# Estimate of adolescent alcohol use in China: a meta-analysis

**DOI:** 10.1186/s13690-016-0157-5

**Published:** 2016-10-25

**Authors:** Yonghua Feng, Ian M. Newman

**Affiliations:** Department of Educational Psychology, University of Nebraska-Lincoln, PO Box 880345, Lincoln, NE 68588-0345 USA

**Keywords:** Alcohol, Adolescents, China

## Abstract

**Objective:**

A profile of adolescent alcohol use for China that specified gender, school type and a consistent definition of alcohol use.

**Method:**

A total of 1,646 papers were identified in the Chinese- and English-language literature published 2007–2015 that reported Chinese adolescent drinking rates. Selection criteria were established *a priori.* Thirty-two papers met all the selection criteria. Five papers were eliminated because they were found to be duplicate reports of the same data.

**Result:**

The resulting sample included 26 papers—24 in Chinese and two in English, 20 describing middle school students, 12 describing high school students, and six describing vocational high school students. Eleven papers described students in more than one type of school. Last 30 day use of alcohol was, as expected, highest among vocational high school students (44.7 % males, 28.8 % females) and drinking rates were higher for high school students (36.5 % males, 21.2 % females) than for middle school students (23.6 % males, 15.3 % females). Meta-regression identified factors associated with differences in drinking rates reported in individual studies as the definition of a drink and whether data were collected by trained personnel. Location appeared important, but its effects were inconsistent across different populations, which suggests that national estimates likely blur regional differences in patterns of alcohol use.

**Conclusion:**

Rates derived from this meta-analysis provide a useful reference for scholars interested in China, alcohol use, adolescents, and patterns of use. The meta-regression analysis suggested practical ways to improve adolescent alcohol surveys in China.

## Background

Between 2003–2005 and 2008–2010 the World Health Organization [1] reported a 36 % increase in alcohol per capita consumption in liters of pure alcohol among adults 15 years and older in China. The per capita consumption of the drinkers aged 15 and above in 2010 was 18.7 L of pure alcohol for males and 7.6 L of pure alcohol for females. A quarter (24.3 %) of the males and 2.5 % of the females participated in heavy episodic drinking, defined as consuming at least 60 g or more of pure alcohol on at least one occasion in the last 30 days [1]. Unfortunately, there are no similar data that can help us understand the developmental trajectory of adolescent alcohol use to adult use. A previous review of papers on Chinese adolescent alcohol use published in China between 1994 and 2007 [2] failed to generate an estimate of alcohol use because of inconsistencies in the definitions of alcohol use and the demographic variables collected.

Accordingly, a research protocol was developed to analyze available literature published between 2007 and 2015 to provide a profile of adolescent alcohol use in China that specified gender, school type, and a consistent definition of alcohol use. Six research questions focused this analysis.

1. What is the drinking rate for Chinese middle school male students in the last 30 days?
2. What is the drinking rate for Chinese middle school female students in the last 30 days?
3. What is the drinking rate for Chinese high school male students in the last 30 days?
4. What is the drinking rate for Chinese high school female students in the last 30 days?
5. What is the drinking rate for Chinese vocational high school male students in the last 30 days?1
6. What is the drinking rate for Chinese vocational high school female students in the last 30 days?

## Method

A research protocol was drafted prior to starting this project. The protocol specified the search strategy, study selection, inclusion and exclusion criteria, data extraction, and meta-analysis.

### Search strategy

This project followed the guidelines of the *Cochrane Handbook for Systematic Reviews of Interventions* [3]. Our interest was the Chinese adolescent drinking rate in the last 30 days. We chose to conduct an online literature search for articles and abstracts published between 2007 and 2015 on four electronic databases: PubMed (http://www.ncbi.nlm.nih.gov/pubmed/advanced), Web of Science (www.webofknowledge.com), China National Knowledge Infrastructure (CNKI) (http://www.cnki.net/), and Wanfang (http://www.wanfangdata.com.cn/). The lists of references in the identified papers were then searched for references to meetings, seminars and other possible sources of information that were not formally published, sometimes referred to as “grey literature”. No additional studies were identified by means of searching the references of the retrieved articles.

The initial search of abstracts in CNKI and WF used the search words: adolescent OR middle school student OR high school student OR middle and high school students AND alcohol use/drinking. The same search words plus Chinese/China were used in search of abstracts in PubMed and in the search of topic in Web of Science. An example of the search strategy for one database (CNKI) is included the Appendix. The selection of the articles was performed by one reviewer. To increase the accuracy, the reviewer performed the search twice for each database. No new references were identified on the second search.

### Inclusion and exclusion criteria

The inclusion criteria established prior to the study were: 1) papers based on actual reported survey data, 2) collected in mainland China, not including Hong Kong, Macau or Taiwan, 3) papers that reported alcohol use in the last 30 days, 4) separately for males and females, 5) and separately for school type: middle school, high school, and vocational high school. The reasons for establishing these criteria are explained below.

### Actual survey data

Actual survey data was essential to avoid descriptions from secondary sources and conclusions presented without clear documentation.

### Mainland China

This review was limited to papers describing alcohol use in mainland China because the shared recent history of all parts of the mainland is different from that of Hong Kong, Macau, and Taiwan.

### Drinking in the last 30 days

This measure was chosen because drinking in the last 30 days was considered more accurate than the drinking in the past year, as it has a lower chance of memory error [4, 5].

### Gender

Differences between male and female drinking rates among adults in China are large [1, 6, 7]. A 2008 multi-province survey using a Chinese version of the US Centers for Disease Control and Prevention’s Youth Risk Behavior Survey [8] confirmed that adolescent drinking rates differed significantly by gender, as did another large-sample provincial study [9].

### School type

School type served as a proxy for age and academic intent. Middle schools typically serve students ages 12–15, and high schools and vocational high schools include students ages 15–18. Vocational high schools educate students who are not preparing for the competitive examination for admission to university but who are preparing to move directly to employment. The statistical difference in alcohol use among students of different school types has been reported in various studies [8, 10–13].

### Outcome variable and moderators

The outcome variable is the drinking rate in the last 30 days for middle, high, and vocational high school males and females. Based on Moher et al’s [14] quality assessment checklists, Groves and Lyberg’s [15] discussion of the theoretical sources of survey error, and the authors’ knowledge of alcohol use and survey research in China, eleven moderators were identified. They were: sample size, a definition of 30 day drinking that specified drinking at least a cup of alcohol, data that was collected by a trained collector, reported response rate, data reported as a number (n) and percentage (%)—which suggested a greater attention to detail, year the study was conducted, geographic location, minority region, county/rural *versus* urban, number of schools surveyed, and authorship (academic author or government report).

### Moderator coding procedure

Sample size was coded as one of three levels: ‘1’ with a sample size at or below the 33.3 percentile; ‘2’ for a sample size between 33.4 and the 66.6 percentile; or ‘3’ with a sample size above the 66.6 percentile. Dummy codes, with ‘1’ for ‘Yes’ and ‘0’ for ‘No’, were used for each of the following: a definition that defined a drinker as drinking one cup of alcohol in the last 30 days, reported data was collected by a trained collector, reported the response rate, results reported in both numbers and percent of alcohol use, minority region identified, and county/rural area identified. For authorship, academic was coded ‘1’, and government was coded ‘0’. The guidelines for identifying minority regions followed the guideline of the Chinese Minority Compact Communities, which identified communities with significant minority populations [16]. Year the study was conducted and the number of surveyed schools were coded as integers. Location was coded into two moderators; first, ‘east’, ‘middle’, and ‘west’, and then ‘south’ and ‘north’. The ‘east’, ‘middle’, and ‘west’ designations followed the National Bureau of Statistics of China definitions [17], and the ‘south’ and ‘north’ were separated by the Qing Mountain and Huai River line, a customary division. Two studies that included samples from more than one region were deleted from the analysis by location [18, 19].

A data extraction plan was developed by the authors, and codes were placed into a Microsoft Excel spreadsheet. Data extraction was performed independently by two advanced graduate students who are fluent in both Chinese and English. The drinking rate was extracted as a rate; the 10 moderator extraction criteria are described above. Initial rater agreement was 97.4 %. Discrepancies were then re-examined and resolved, resulting in 100 % agreement.

### Meta-analysis

A DerSimonian and Laird random-effect model was used to estimate the drinking rates with the inverse-variance weighting scheme. The DerSimonian-Laird model is recommended when there is no reason to assume that studies have identical effect [20], and it has performed well in different scenarios in a simulation study [21]. The inverse-variance weighting scheme assigns heavier weights to larger studies [22].

### Publication bias

Funnel plots were used to visually examine the symmetry of the outcome variables. It is recommended that analyses include at least 10 studies [3]. Egger’s linear regression test of the intercept to quantify the bias captured by the funnel plot was used to test for significance [23]. Only six papers describing vocational high school students met our selection criteria, insufficient to meet Egger’s minimum of 10. Consequently we used the trim and fill method proposed by Duval and Tweedie [24].

### Measure of heterogeneity

*I* ^2^, the percentage of between-study variance due to systematic heterogeneity rather than chance [25], was used to estimate the heterogeneity among studies. A value of 0 % indicated no observed heterogeneity, and higher values indicated larger heterogeneity, with 0–25 % as low, 26–50 % as moderate and 51–75 % as high [25]. Cochran’s *Q* test was also used to determine whether the differences in drinking rate estimates across studies were larger than expected by chance. *Q* has a chi-square distribution of k-1, where k is the number of effect sizes. A significant *Q* value indicates heterogeneity among reviewed studies.

### Sensitivity analysis

A sensitivity analysis was conducted by omitting one study at a time in order to interpret the significance of the heterogeneity and to understand the impact of individual studies on the overall results.

### Meta-regression

Meta-regression with the maximum likelihood estimation was used to explore factors associated with the true between-study variance (*I* ^*2*^). *R* ^*2*^ is the measure of how much the between-study variance is explained by the moderator(s). It is suggested the analysis should include at least 10 studies [3], and the ratio of the number of moderators to the number of studies should not be larger than 1/10 [26]. The log-transformed value of ratio outcome variable was used to generate a symmetric scale with a symmetric confidence interval [3]. Bivariate meta-regression analysis was first used to examine the relationship between a moderator and the drinking rate, and then a hierarchal regression was used that added all the significant moderators into the model. The significance level was set at .10 for the bivariate meta-regression, because the statistical power is reduced due to the limited number of studies. This level of significance has been used in previous systematic reviews [27, 28]. Bonferroni correction was used to decide the significant level of moderators in the hierarchal meta-regression.

### Subgroup analysis

Subgroup analysis was used for the categorical moderators that were significantly associated with the heterogeneity identified in the hierarchical meta-regression and the bivariate meta-regression if only one moderator was identified. Random-effect model was used to estimate the pooled drinking rate for subgroups. The *Q* value for between groups, similar to the between group variance in ANOVA, was reported along with the *p* value.

Comprehensive Meta-analysis Professional Version 3 was used for the data analyses. It has the advantage of automatically coding dummy variables from categorical data.

## Result

A total of 1,646 papers were initially identified. Based on a careful reading of the title and abstracts, 201 full-text papers (14 in English and 187 in Chinese) were selected for full review. Thirty-one papers met all the selection criteria. There were five sets of papers presenting duplicate data. In the case of the duplicate reports, the least complete paper was eliminated. The total number of papers used in the analysis was 26 papers.

**Fig. 1:**
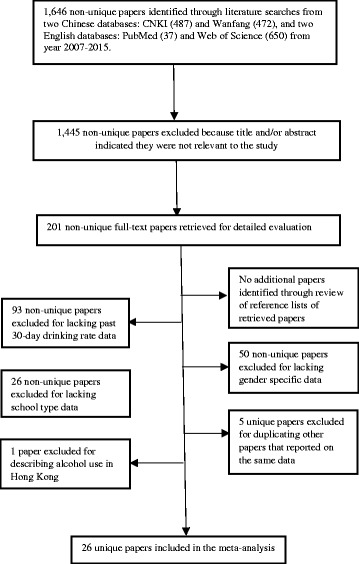
Flowchart of selection of studies for inclusion in meta-analysis

The reasons for excluding papers from the sample of 201 were: no 30-day drinking rate data (93 papers); no gender specific data (50 papers); no school specific data (26 papers); and one paper described drinking in Hong Kong. A flowchart of the selection of studies for inclusion in the analysis is presented in Fig. 1.

The resulting sample included 26 papers, 24 in Chinese and two in English, 20 describing drinking by middle school students, 12 describing drinking by high school students, and six describing drinking by vocational high school students. Eight papers described students in more than one type of school.

The samples described in these papers came from 12 of China’s 22 provinces, two of the four municipalities, and two of the five autonomous regions (Table 1).

**Table 1:** Characteristics of the sample of the included studies

Reference	Male drinking rate	Female drinking rate	Sample size	School number	Year of study	Response level	Collector	Minority region	Definition	Authorship	Case number and %	Rural	City/province
Middle school													
Lin et al., 2010 [12]	21.1	18.9	1,338	NA	2008	99.88	Yes	No	No	No	No	No	Fuzhou, Fujian
An et al., 2013 [18]	16.4	12.2	1,985	8	2010	94.20	Yes	No	Yes	No	Yes	No	Shenyang, Liaoning, Guangzhou, Guangdong
Lu et al., 2015 [19]	18.2	13.9	6,575	67	2013	96.50	Yes	No	Yes	Yes	Yes	No	Beijing, Shanghai, Guangzhou
Aximu et al., 2007 [29]	28.5	20.5	3,575	NA	NA	NA	Yes	Yes	No	No	No	No	Three cities across Xinjiang
Bao et al., 2014 [34]	49.6	27.6	1,608	4	2011	98.50	Yes	No	No	Yes	No	No	Xi’an, Shaan Xi
Huang & Wang, 2010 [35]	33.1	21.9	1,773	8	2008	99.16	Yes	No	Yes	No	Yes	No	Haidian district, Beijing
H. Liu & Jia, 2010 [36]	42.3	23	1,933	8	NA	94.02	Yes	No	Yes	No	Yes	No	Tianjin City
Y. Liu et al., 2013 [37]	21.3	15.2	2,005	NA	2008	NA	No	No	No	Yes	Yes	No	Bejing
Ruan et al., 2009 [38]	30.7	19.7	4,983	22	2008	99.45	Yes	Yes	Yes	No	Yes	No	3 counties and 3 cities across Guangxi
Tang et al., 2015 [39]	18.1	11.5	2,527	18	NA	96.04	Yes	No	Yes	No	No	No	Shanghai City
C. Wu & Xie, 2009 [40]	23.5	21.1	1,345	8	2008	95.95	Yes	No	No	No	No	No	Zaozhuang, Shandong
S. Wu & Wang, 2011 [41]	23.8	23.9	785	4	2006	96.70	Yes	No	No	Yes	Yes	No	Guangzhou, Guangdong
G. Yu et al., 2010 [42]	21.8	4.1	859	6	NA	97.80	No	Yes	No	Yes	No	Yes	Taojiang County, Henan
J. Yu et al., 2010 [43]	20.3	17.4	456	2	2008	98.91	Yes	No	Yes	No	Yes	Yes	Tonglu Town, Zhejiang
L. Zhang & Zhang, 2015 [44]	31.9	18.6	957	5	2013	NA	Yes	Yes	No	Yes	Yes	Yes	Xiangxi, Hunan
R. Zhang et al., 2013 [45]	20.2	13.7	2,939	17	NA	NA	Yes	No	No	No	No	No	Three cities across Zhejiang Province
X. Zhang et al., 2011 [46]	19.1	13.5	1,802	25	NA	98.00	Yes	No	No	No	Yes	No	Hangzhou, Zhejiang
Y. Zhang et al., 2014 [47]	12.9	12.6	1,174	7	2013	NA	Yes	No	Yes	No	Yes	No	Changzhou, Jiangsu
Zhong et al., 2007 [48]	24.7	7.2	5,158	NA	2004–2005	NA	Yes	No	No	No	No	No	Four cities across Henan
Zhu et al., 2009 [49]	12.2	7.7	1,427	4	2008	97.40	No	Yes	No	No	No	No	Zhangye, Gansu
High school													
Zhao & Lv, 2013 [33]	13.3	6.3	1,768	6	NA	98.20	No	No	Yes	Yes	No	No	Taiyuan, ShanXi
Ruan et al., 2009 [38]	43.7	21.1	5,072	21	2008	99.45	Yes	Yes	Yes	No	Yes	No	3 counties and 3 cities across Guangxi
Tang et al., 2015 [39]	25.2	14.6	1,050	18	NA	96.04	Yes	No	Yes	No	No	No	Shanghai City
C. Wu & Xie, 2009 [40]	30.3	36.7	1,988	8	2008	95.95	Yes	No	No	No	No	No	Zaozhuang, Shandong
J. Yu et al., 2010 [43]	32.2	15.3	408	2	2008	98.91	Yes	No	Yes	No	Yes	Yes	Lutong Town, Zhejiang province
Y. Zhang et al., 2014 [47]	29.1	16	826	4	2013	NA	Yes	No	Yes	No	Yes	No	Changzhou, Jiangsu
Zhong et al., 2007 [48]	40.4	18	7,627	NA	2004-2005	NA	Yes	No	No	No	No	No	Four cities across Henan
Zhu et al., 2009 [49]	49.4	28	1,855	4	2008	97.40	Yes	Yes	No	No	No	No	Zhangye, Gansu
Shao et al., 2013 [50]	37.3	23.4	8,329	NA	2008–2010	NA	Yes	No	Yes	No	Yes	No	Seven cities across Liaoning
D. Wu et al., 2008 [51]	36	25.8	1,011	4	2007	99.70	Yes	No	No	No	No	No	Shenzhen City
Xu et al., 2014 [52]	71.7	53.6	1,440	4	2012	97.60	Yes	No	No	Yes	Yes	No	Xi’an, Shaan Xi
L. Zhang & Ma, 2013 [53]	39.6	16	437	1	NA	95.00	No	Yes	No	Yes	No	No	Shihezi, Xinjiang
Vocational high school													
Ruan et al., 2009 [38]	64.5	34.4	2,420	11	2008	99.45	Yes	Yes	Yes	No	Yes	No	3 counties and 3 cities across Guangxi
Tang et al., 2015 [39]	39.4	32.9	1,034	10	NA	96.04	Yes	No	Yes	No	No	No	Shanghai City
J. Yu et al., 2010 [43]	48.4	35.8	228	1	2008	98.91	Yes	No	Yes	No	Yes	Yes	Lutong Town, Zhejiang
Y. Zhang et al., 2014 [47]	25.1	18.7	601	3	2013	NA	Yes	No	Yes	No	Yes	No	Changzhou, Jiangsu
Shao et al., 2013 [50]	51.9	32.7	3,572	NA	2008–2010	NA	Yes	No	Yes	No	Yes	No	Seven cities across Liaoning
Liang & Zeng, 2013 [54]	39.9	20.9	1,013	2	NA	96.10	Yes	No	No	Yes	No	No	Chaozhou, Guangdong

**Fig. 2:**
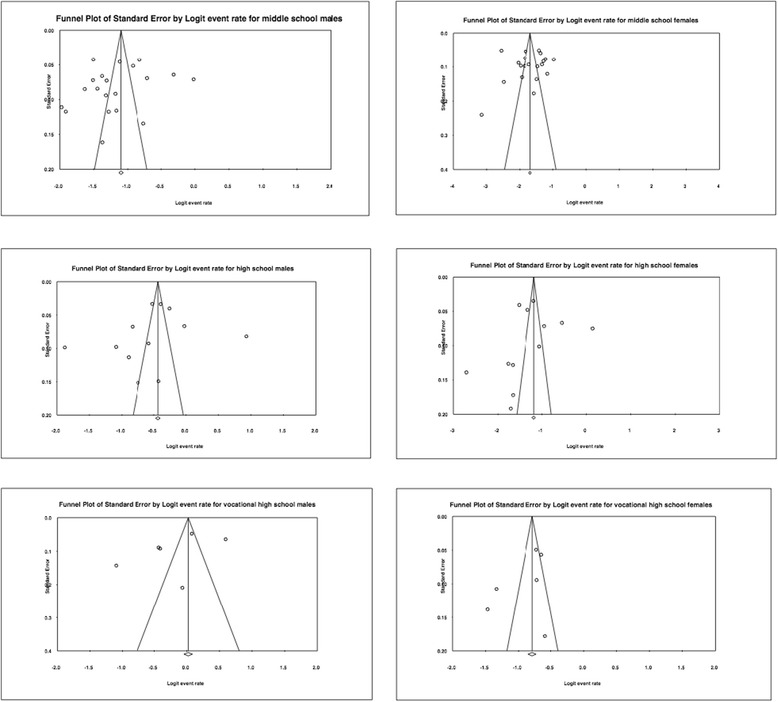
Funnel plots of drinking rate in the last 30 days among adolescents in China using the DerSimonian-Laird random-effect model

Funnel plots for male and female middle, high, and vocational high school students are symmetric (Fig. 2). Following the results of Egger’s Test for small study effects, the six tests showed the non-significant result (seen in Table 2), which indicates no publication bias. By using the trim and fill method for vocational high school students, there was no evidence of publication bias.

**Table 2:** Egger’s two tail test for middle, high, and vocational high male and female students’ drinking rate in the last 30 days

	*t*	*df*	*SE*	*p*
Middle school male students	.857	18	4.126	.403
Middle school female students	.017	18	3.784	.987
High school male students	.665	10	4.827	.522
High school female students	.311	10	4.830	.762
Vocational high school male students	1.34	4	5.655	.250
Vocational high school female students	1.38	4	3.138	.238

*N* = 20 for middle school males and females, *N* = 12 for high school males and females, *N* = 6 for vocational high school males and females

**Fig. 3:**
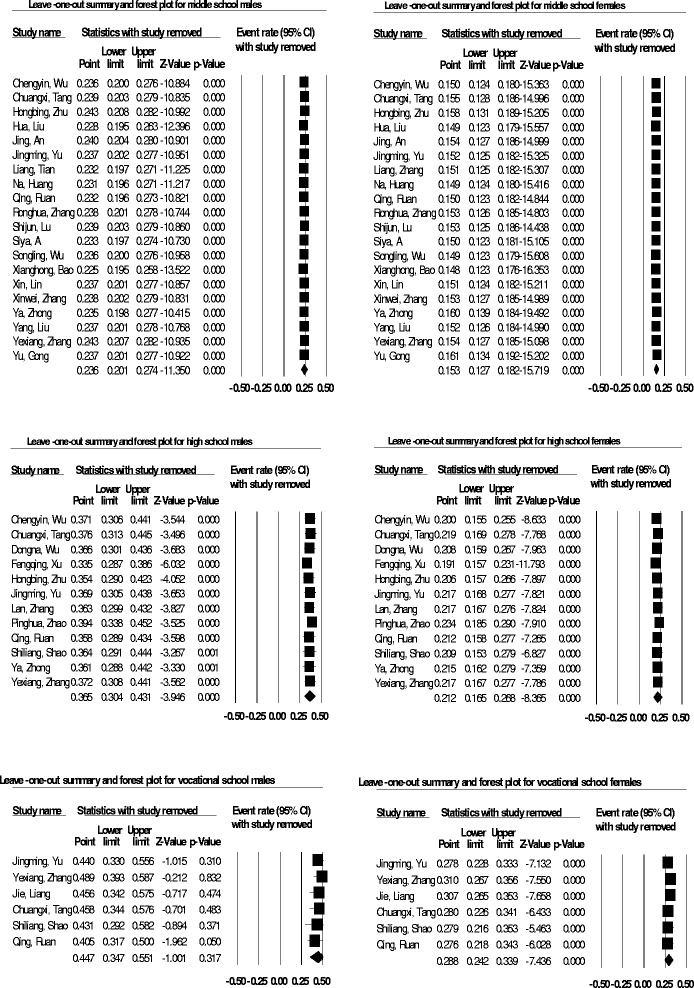
Sensitivity analysis statistic summary and forest plots among adolescents in China using the DerSimonian-Laird random-effect model

The result of the sensitivity analysis is shown in Fig. 3 for male and female middle, high, and vocational high school students are consistent. This analysis showed no single study having a significant effect on the overall result.

Table 3 shows the pooled drinking rate estimates. The meta-regression results and the sub-group analyses are seen in Tables 4 and 5 respectively.

**Table 3:** Pooled estimates of drinking rate (last 30 days) using the DerSimonian-Laird random-effect models, data from China 2007–2015

subgroup	No. of studies	Range of prevalence (%)	Pooled prevalence (%)	95 % CI (%)	*I* ^*2*^ (%)
	Middle school students drinking rates				
Male	20	12.2, 49.6	23.6	20.1, 27.4	97.6***
Female	20	4.1,27.6	15.3	12.7, 18.2	97.1***
	High school students drinking rates				
Male	12	13.3, 71.7	36.5	30.4, 43.1	98.3***
Female	12	6.3, 53.6	21.2	16.5, 26.8	98.3***
	Vocational high school students drinking rates				
Male	6	25.1, 64.5	44.7	34.7, 55.1	97.4***
Female	6	18.7,34.4	28.8	24.2, 33.9	91.6***

*N* = 20 for middle school males and females, *N* = 12 for high school males and females, *N* = 6 for vocational high school males and females. ***p < .001, for Q-test p-value

**Table 4:** Factors associated with the heterogeneity of drinking rate (last 30 days) estimates among Chinese middle and high school students using meta-regression for four outcomes reported in 10 or more studies from 2007–2015

Outcome variables	Moderator	Bivariate *b*	*R* ^*2*^%	Hieratical *b*	*R* ^*2*^%
Middle school students’ drinking rate					
Male	Location (north/south)	.368*	15		
Female	Location (east to middle)	.741**	28	.589*	50
	Location (west to middle)	.776*		.804*	
	Collector (Yes)	.790**	28	.727**	
High school students’ drinking rate					
Male	Location (east to middle)	.422	49	-.106	80
	Location (west to middle)	1.29**		1.05***	
	Definition (Yes)	-.675*	28		
	Collector (Yes)	.729*	19	1.117***	
Female	Location (east to middle)	.779*	34	.474	80
	Location (west to middle)	.805*		.867**	
	Collector (Yes)	1.043*	33	.959**	
	Definition (Yes)	-.794*	35	-.588*	

*N* = 20 for middle school males and females, *N* = 12 for high school males and females, *N* = 6 for vocational high school males and females. ***p < .001, **p < .01, *p < .1. The moderator of authorship for high school female students was significantly associated with drinking rate, it also has a strong correlation (*r* = −.775, *p* = .003) with the moderator of trained data collector. To deal with the collinearity issue, the moderator of authorship was removed in the hieratical meta-regression analysis. Bonferroni correction applied on the significance level of .1: the significant level changed to .05 for 2 moderators, .033 for 3 moderators; Bonferroni correction applied on the significant level of .01, the significant level changed to .005 for 2 moderators, .003 for 3 moderators; Bonferroni correction applied on the significant level of .001, the significant level changed to .0005 for 2 moderators, and .0003 for 3 moderators

**Table 5:** The pooled estimates of drinking rates (last 30 days) among different groups by using DerSimonian-Laird random-effect model

Outcome variable	Subgroup	Number of studies	Pooled estimate	95 % CI	*I* ^*2*^	*Q*_between_
Middle school male students’ drinking rate						
	Location south	11	21.8	18.8, 25.2	93.9	2.47
	Location north	7	28.7	21.0, 37.8	98.3	
Middle school female students’ drinking rate						
	Location east	14	16.6	14.6, 18.9	92.0	3.26
	Location middle	3	8.5	3.9, 17.4	96.8	
	Location west	3	17.0	9.8, 27.8	97.7	
	Collector (Yes)	17	16.8	13.8, 20.2	95.3	4.0*
	Collector (No)	3	8.1	3.9, 16.1	97.2	
High school male students’ drinking rate						
	Location east	7	33.4	28.9, 38.2	94.6	5.05*
	Location middle	2	24.4	7.0, 58.1	99.5	
	Location west	3	54.2	35.7, 71.6	98.1	
	Collector (Yes)	10	39.3	33.5, 45.5	97.9	.956
	Collector (No)	2	24.0	7.0, 56.7	98.5	
	Definition (Yes)	6	29.1	21.7, 37.8	98.4	5.024*
	Definition (No)	6	44.6	34.1, 55.5	98.2	
High school female students’ drinking rate						
	Location east	7	21.5	17.3, 26.2	95.8	2.89
	Location middle	2	10.9	3.7, 28.1	98.5	
	Location west	3	30.8	14.9, 53.1	98.7	
	Collector (Yes)	10	24.1	18.9, 30.2	98.3	3.63*
	Collector (No)	2	10.1	3.9, 23.9	96.2	
	Definition (Yes)	6	15.4	11.5, 20.1	98.9	5.68*
	Definition (No)	6	28.4	18.6, 40.8	96.4	

*N* = 20 for middle school males and females, *N* = 12 for high school males and females, *N* = 6 for vocational high school males and females. *p < .1. Only studies that contained the moderator information are included in the group comparisons. For middle school students, the location for two studies that included samples from more than one region were deleted from the comparison [18, 19]

### Middle school male students

The pooled estimates for the drinking rate in the last 30 days among middle school male students was 23.6 %, 95 % CI [20.1, 27.4]. There was significant heterogeneity (*I* ^*2*^ = 97.6) among the 20 selected studies. The moderator location (‘north’ vs. ‘south’) was significantly associated with the logit drinking rate of middle school male students, and explained 15 % of the heterogeneity. In the subgroup analysis, the pooled drinking rate for middle school male students was 21.8 % in south, and 28.7 % in north. However, the group difference is not significant (*Q* (1) = 2.47, *p* = .12).

### Middle school female students

The pooled estimates for the drinking rate in the last 30 days among middle school female students was 15.3 %, 95 % CI [12.7, 18.2]. There was significant heterogeneity (*I* ^*2*^ = 97.1) among the selected 20 studies. The moderator location (‘east’, ‘middle’, ‘west’) and trained data collector were significantly associated with the logit drinking rate of middle school female students in the bivariate meta-regression. In the hierarchal meta-regression analysis, the two moderators were still significant after Bonferroni correction and explained 50 % of the heterogeneity. In the subgroup analysis, the pooled drinking rate for middle school female students in east China was 16.6 %, 95 % CI [14.6, 18.9], in middle China was 8.5 %, 95 % CI [3.9, 17.4], and in west China was 17.0 %, 95 % CI [9.8, 27.8]. However, the group difference was not significant (*Q*(2) = 3.259, *p* = .20). The pooled drinking rate for middle school female students with trained data collectors was 16.8 %, 95 % CI [13.8, 20.2], and without trained data collectors it was 8.1 %, 95 % CI [3.9, 16.1]. This difference was significant (*Q*(1) = 4.0, *p* = .046).

### High school male students

The pooled estimates for the drinking rate in the last 30 days among high school male students was 36.5 %, 95 % CI [30.4, 43.1]. There was significant heterogeneity (*I* ^*2*^ = 98.3) among the selected 12 studies. The moderator location (‘middle’, ‘west’), a definition of drinking that specified at least one cup, and trained data collectors were significantly associated with the logit drinking rate of high school male students. In the hierarchal meta-regression analysis, the moderator definition that specified one cup was not a significant factor, and the other two moderators, location (‘middle’, ‘west’) and trained data collectors, were still significant after Bonferroni correction and explained 80 % of the heterogeneity. In the subgroup analysis, the pooled drinking rate for high school male students in east China was 33.4 %, 95 % CI [28.9, 38.2], in middle China was 24.4 %, 95 % CI [7.0, 58.1], and in west China was 54.2 %, 95 % CI [35.7, 71.6]. The difference was significant (*Q*(2) = 5.05, *p* = .08). The pooled drinking rate for high school male students with trained data collectors was 39.3 %, 95 % CI [33.5, 45.5] and without trained data collectors was 24.0 %, 95 % CI [7.0, 56.7]. The difference was not significant (*Q*(1) = .96, *p* = .328).

### High school female students

The pooled estimates for the drinking rate in the last 30 days among high school female students was 21.2 %, 95 % CI [16.5, 26.8]. There was significant heterogeneity (*I* ^*2*^ = 98.3) among the 12 selected studies. The moderator of location (‘middle’, ‘west’), trained data collectors, and the one cup definition were significantly associated with the logit drinking rate of high school female students. In the hierarchal meta-regression analysis, the three moderators were still significant after Bonferroni correction and explained 80 % of the heterogeneity. In the subgroup analysis, the pooled drinking rate for high school female students in east China was 21.5 %, 95 % CI [17.3, 26.2], in middle China was 10.9 %, 95 % CI [3.7, 28.1], and in west China was 30.8 %, 95 % CI [14.9, 53.1]. However, there was not a significant group difference (*Q*(2) = 2.89, p = .236). The pooled drinking rate for high school female students with trained data collectors was 24.1 %, 95 % CI [18.9, 30.2] and without trained data collectors was 10.1 %, 95 % CI [3.9, 23.9]. The difference was significant (*Q*(1) = 3.63, *p* = .057). The pooled drinking rate for high school female students from studies that included the one cup definition was 15.4 %, 95 % CI [11.5, 20.1], and from studies without the one cup definition was 28.4 %, 95 % CI [18.6, 40.8]. The difference was significant (*Q*(1) = 5.68 *p* = .017).

### Analyses results for vocational high school students

The pooled estimates for the drinking rate in the last 30 days among vocational high school male students was 44.7 %, 95 % CI [34.7, 55.1] and for female students was 28.8 %, 95 % CI [24.2, 33.9] with large heterogeneity (*I* ^*2*^ = 97.4; *I* ^*2*^ = 91.6 respectively). Only six papers met our selection criteria, less than the 10 recommended for funnel plot and meta-regression analysis so they were not completed.

## Discussion

### Drinking rates

This study is a first comprehensive review of the published literature on Chinese adolescents’ alcohol drinking rates, by gender and school type. Of the 1,646 papers initially identified, 20 papers on middle school student’s alcohol use met the *a priori* selection criteria, 12 papers on high school students alcohol use met the criteria, and six papers on vocational high school students alcohol use met the criteria. The pattern of drinking was, as expected, highest among vocational high school students (44.7 % males, 28.8 % females) and drinking rates were higher for high school students (36.5 % males, 21.2 % females) than for middle school students (23.6 % males, 15.3 % females). In all three types of schools drinking rates for males were higher than drinking rates for females. While there have been large sample studies of adolescent alcohol use in China in the years covered by this study [6, 8, 9], none presented a profile of student alcohol use that specified gender, school type and a consistent definition of alcohol use.

These results reflect the best estimate of adolescent alcohol use in the last 30 days for our selected demographic variables. Although none of our analyses showed that the heterogeneity affected the resulting adolescent alcohol use estimates, nevertheless we have to be cautious when using these estimates given the range of drinking rates found in the studies we examined and the significant I^2^. Since this is the first meta-analysis of Chinese adolescent drinking studies, it was important to identify the potential sources for this heterogeneity. Eleven moderators were examined in this meta-regression, and three of them (location, data collectors, and definition of “drinking”) were associated with heterogeneity. These three are discussed in detail below. Our analysis couldn’t assess many other factors that were possibly associated with heterogeneity, such as the fact that school-based surveys are new in China. Little is known about how the samples were selected, how the surveys were administered, and how the actual survey experience was perceived by the young people completing the questionnaires. We know little about whether the students participated voluntarily and how anonymity and confidentiality might have been protected. We also know little about the selection of Chinese words and their translation and interpretation. The higher rates of alcohol use reported by vocational high school students is understandable. However, these students likely represent a wider socioeconomic range than those going to traditional high schools. The number of studies describing vocational high school students in this analysis was too few to conduct meaningful moderator analyses. The vocational high school student population, because of its diversity, likely deserves closer analysis in the future.

The results of our moderator analysis did suggest specific variables that need to be considered in interpreting survey results. These include the geographic location of the surveys, the training of the data collectors, and the way an alcohol drink was described in the survey question.

### Location

Though there were no statistically significant differences found among east, middle, and west China, and south and north China, the moderator of location did significantly explain some of the heterogeneity among collected studies. This suggests that national estimates will likely fail to accurately describe regional differences. Future studies will need to carefully consider regional sampling. Ji [8] in discussing the results of the 2008 Youth Risk Behavior Study also identified this concern. While China’s recognized ethnic minority groups make up less than 10 % of the nation’s total population, they are concentrated in certain regions, and this could significantly affect regional alcohol survey results. For example, the Hui, who are predominantly followers of Islam, with an estimated population of 10.5 million, are located mainly in the northwestern provinces.

### Trained data collectors

The lower rates recorded among females when data was collected with trained personnel suggests the importance of trained data collectors in all future surveys. Because surveys of adolescent alcohol use are often conducted in schools, it is assumed that teachers can serve as data collectors. This analysis underscored the importance of using trained data collectors.

### Defining ‘drinking’ in alcohol surveys

Some researchers defined current alcohol use as those who drank at least once in the last 30 days, including a sip [10, 29, 30]. Other researchers specified that a current alcohol user is one who drank at least a cup of alcohol in the last 30 days [8, 11, 31–33]. This study found fewer students reported drinking in the last 30 days when the question specified drinking at least a cup. Open access to alcohol (alcohol usually served with meal) provides more opportunities for adolescents to sip rather than ‘drink’ alcohol. Further study should consider the effects of the definition of drinking on reported drinking rates and the minimum ABV (alcohol by volume) to define an alcohol drink.

### A further complication: defining ‘alcohol’ in adolescent alcohol surveys in China

None of the papers in this analysis defined alcohol by ABV.

In China, there is a greater variety of alcohol consumed in a greater number of ways than perhaps researchers have encountered in the West, and this presents an interesting challenge for estimating adolescent alcohol use by means of alcohol survey questions that have been developed in the West. In addition to the type of alcohol that is drunk as a beverage/intoxicant, there are a number of types of alcohol regularly consumed as medicine, as food, and as cooking ingredients in other dishes. For example, there is a category of low-alcohol rice wine (*mi jiu*) that includes both liquid beverages and fermented porridges (*jiu niang, lao zao*) that is consumed by people of all ages as part of a normal diet. The ABV of both homemade and commercial *mi jiu* deserves some attention, because of how frequently *mi jiu, jiu niang and lao zao* are served. The ABV of *huang jiu* (yellow wine) and *liao jiu* (cooking wine) also deserve attention. It may be, as most people tell us, that the ABV of these comestible and cooking alcohols is too low to worry about, but this belief about low ABV alcohols needs to be confirmed. Whether a survey instrument defines the minimum ABV that qualifies a food/beverage as ‘alcohol’ and whether a survey instructs participants to count medicinal, comestible and cooking alcohol affects the resulting alcohol use rates that are reported.

### Limitation

The number of papers was insufficient to run all the moderators at the same time for middle and high school males and females. This limitation possibly resulted in exaggerating the precision of the moderators. There were not enough papers to run a meta-regression for vocational high school males and females and to examine the potential moderators associated with the large heterogeneity in vocational high school student drinking rates. Nevertheless, this study gives a first review of the recent studies of rates of adolescent alcohol use in China. As such it presents cautious guidance to those who seek a better understanding of alcohol use throughout the lifespan and who plan future surveys of adolescent alcohol use.

## Conclusion

The World Health Organization has regularly estimated for China the alcohol use rate for persons 15 years of age and older [1]. They have not estimated alcohol use by younger members of the population. This information would be helpful in understanding the development of adult drinking patterns.

There have been many papers published that describe adolescent alcohol use in China, and the reported rates of alcohol use have differed greatly. The variations are the result of different definitions of alcohol, differences in the survey questions asked, different data gathering methods, samples of adolescents from different types of schools, and from different regions, and the time of year in which the alcohol use survey is conducted. This paper is the first attempt to identify drinking rates among adolescents of middle-school and high-school age, using a regularized meta-analysis technique. The results suggest a best estimate of last-30-day drinking rates among adolescents, which appeared to relate logically to the adult patterns of alcohol use reported by WHO. The pattern of drinking was, as expected, highest among vocational high school students (44.7 % males, 28.8 % females) and drinking rates were higher for high school students (36.5 % males, 21.2 % females) than for middle school students (23.6 % males, 15.3 % females). In all three types of schools drinking rates for males were higher than drinking rates for females. This analysis identified a number of ways that future studies of adolescent drinking could be improved to generate more accurate estimates.

## Appendix

China National Knowledge Infrastructure was searched in spring 2016, the searching strategy is as follow:

1. Went to the search link of CNKI: http://www.cnki.net/
2. Went to advance search in CNKI
3. Selected journal articles
4. Selected ‘Theme’ (Theme finds more articles than abstract, so we decided to use theme)
5. Selected year 2007–2015
6. Key terms and number of searched studies
  - Adolescent (青少年)AND Alcohol Use/ drinking (饮酒) (retrieved 254 articles, after reading the articles’ title and abstracts, download 100 full texts)
  - Middle School Students (初中生)AND Alcohol Use/drinking (饮酒)(retrieved 50 articles, after reading the articles’ title and abstracts, download 9 full texts)
  - High School Students (高中生) AND Alcohol Use/drinking (饮酒) (retrieved 56 articles, after reading the articles’ title and abstracts, download 20 full texts)
  - Middle School and High School Students (中学生) AND Alcohol Use/drinking (饮酒) (retrieved 127 articles, after reading the articles’ title and abstracts, download 19 $\overleftarrow{\mathrm{full}\kern.3em \mathrm{texts}\Big)}$

## Abbreviations

ABV: Alcohol by volume
CI: Confidence interval
CNKI: China National Knowledge Infrastructure index of publications
WF: Wanfang index of publications
